# TWEAK and Fn14 expression in the pathogenesis of joint inflammation and bone erosion in rheumatoid arthritis

**DOI:** 10.1186/ar3294

**Published:** 2011-03-24

**Authors:** Anak ASSK Dharmapatni, Malcolm D Smith, Tania N Crotti, Christopher A Holding, Cristina Vincent, Helen M Weedon, Andrew CW Zannettino, Timothy S Zheng, David M Findlay, Gerald J Atkins, David R Haynes

**Affiliations:** 1 Discipline of Anatomy and Pathology, School of Medical Sciences, University of Adelaide, Frome Road, Adelaide, SA 5005, Australia; 2 Rheumatology Research Unit, Repatriation General Hospital, Daws Road, Adelaide, SA 5041, Australia; 3 Myeloma Research Laboratory, Bone and Cancer Laboratories, Division of Haematology, Institute of Medical & Veterinary Science, Frome Road, Adelaide, SA 5005, Australia; 4 Immunology, Biogen Idec Inc., Cambridge Centre, Cambridge, MA 02142, USA; 5 Bone Cell Biology Group, Discipline of Orthopaedics and Trauma, University of Adelaide, Frome Road, Adelaide, SA 5005, Australia; 6 Hanson Institute, Frome Road, Adelaide, SA 5005, Australia

## Abstract

**Introduction:**

TNF-like weak inducer of apoptosis (TWEAK) has been proposed as a mediator of inflammation and bone erosion in rheumatoid arthritis (RA). This study aimed to investigate TWEAK and TWEAK receptor (Fn14) expression in synovial tissue from patients with active and inactive rheumatoid arthritis (RA), osteoarthritis (OA) and normal controls and assess soluble (s)TWEAK levels in the synovial fluids from patients with active RA and OA. Effects of sTWEAK on osteoclasts and osteoblasts were investigated *in vitro.*

**Methods:**

TWEAK and Fn14 expression were detected in synovial tissues by immunohistochemistry (IHC). Selected tissues were dual labelled with antibodies specific for TWEAK and lineage-selective cell surface markers CD68, Tryptase G, CD22 and CD38. TWEAK mRNA expression was examined in human peripheral blood mononuclear cells (PBMC) sorted on the basis of their expression of CD22. sTWEAK was detected in synovial fluid from OA and RA patients by ELISA. The effect of sTWEAK on PBMC and RAW 264.7 osteoclastogenesis was examined. The effect of sTWEAK on cell surface receptor activator of NF Kappa B Ligand (RANKL) expression by human osteoblasts was determined by flow cytometry.

**Results:**

TWEAK and Fn14 expression were significantly higher in synovial tissue from all patient groups compared to the synovial tissue from control subjects (*P *< 0.05). TWEAK was significantly higher in active compared with inactive RA tissues (*P *< 0.05). TWEAK expression co-localised with a subset of CD38^+ ^plasma cells and with CD22^+ ^B-lymphocytes in RA tissues. Abundant TWEAK mRNA expression was detected in normal human CD22^+ ^B cells. Higher levels of sTWEAK were observed in synovial fluids isolated from active RA compared with OA patients. sTWEAK did not stimulate osteoclast formation directly from PBMC, however, sTWEAK induced the surface expression of RANKL by human immature, STRO-1^+ ^osteoblasts.

**Conclusions:**

The expression of TWEAK by CD22^+ ^B cells and CD38^+ ^plasma cells in RA synovium represents a novel potential pathogenic pathway. High levels of sTWEAK in active RA synovial fluid and of TWEAK and Fn14 in active RA tissue, together with the effect of TWEAK to induce osteoblastic RANKL expression, is consistent with TWEAK/Fn14 signalling being important in the pathogenesis of inflammation and bone erosion in RA.

## Introduction

TWEAK (TNF-like weak inducer of apoptosis) is a recently described member of the TNF superfamily. It is reported to exert a variety of biological effects through ligation with its receptor, Fn14. The biological effects of TWEAK include induction of pro-inflammatory cytokines, modulation of the immune response and angiogenesis, stimulation of apoptosis and regulation of tissue repair and regeneration [1,2]. The pro-inflammatory effects of TWEAK/Fn14 signalling are mediated by several signalling cascades, including NF-B and the mitogen-activated protein kinases (MAPK), ERK1/2, JNK1/2 and p38 [3]. TWEAK induces the production of a large number of pro-inflammatory molecules, such as matrix metalloproteinase (MMP1), IL-6, IL-8, MCP-I and Regulated upon Activation Normal T Cell Expressed and Secreted (RANTES) by synoviocytes and fibroblasts, as well as ICAM-1, E-selectin, IL-8, and MCP-1 by endothelial cells [4]. The majority of these cytokines are induced by TWEAK/Fn14 induction of the NF-κβ signalling pathway [3,5]. The pro-inflammatory effects of TWEAK are seen in various cell types including glomerular mesangial cells [6], human umbilical vein endothelial cells (HUVEC) [7], human gingival fibroblasts [8], human dermal fibroblasts, synoviocytes [9], chondrocytes, and fibroblasts [2].

Recent reports from us [10] and others [11] are consistent with TWEAK being a key mediator of joint pathology in murine RA models and in human RA [12,13]. Specifically, recombinant TWEAK enhanced the production of MCP-1 and MIP-2 by synovial cells from collagen induced arthritis (CIA) mice *in vitro*, while the addition of TWEAK monoclonal antibody ameliorated paw swelling, synovial proliferation and inflammatory cell accumulation in CIA [10,11]. A role for TWEAK has been described in human RA, where TWEAK induced the proliferation of synovial fibroblasts and increased the production of inflammatory cytokines and chemokines, as well as the expression of ICAM-1 [12]. High serum levels of TWEAK, TNF-α and IL-6 were seen in RA patients as compared to normal controls [13]. Moreover, serum TWEAK levels correlated with the disease activity score (DAS28) in RA patients and high serum TWEAK levels demonstrated a correlation with short-term response to etanercept treatment [13]. Higher levels of TWEAK were found in RA compared to psoriatic synovium [14]. In the current study we examine TWEAK expression in a larger group of patient-derived samples that encompassed active and inactive RA, osteoarthritic (OA) and normal patients. In addition, levels of soluble (s) TWEAK in the synovial fluids of active RA compared with OA patients were determined.

Pertinent to the pathogenesis of cartilage and bone loss in RA, TWEAK has been demonstrated to promote bone and cartilage destruction through inhibition of chondrogenesis, osteogenesis and the induced production of matrix metalloproteinase (MMP)-3 [10,15]. We have recently described a role for TWEAK in human osteoblast differentiation [16] and Polek *et al. *have proposed its role in osteoclastogenesis [17]. In a more recent study, we demonstrated a significant relationship between serum TWEAK levels and bone erosion markers in patients with bone destructive multiple myeloma [18]. In addition, we have shown that TWEAK, alone and together with TNF-α, induces the expression by osteoblasts and osteocytes, *in vitro *and in *ex vivo *bone, of the bone formation inhibitor, sclerostin, via a MAPK-dependent pathway [16] suggesting a means by which TWEAK may inhibit bone formation during inflammatory bone remodeling. These findings suggest that TWEAK may modulate the bone erosion associated with several diseases, such as rheumatoid arthritis (RA) and multiple myeloma. The present study extends these findings by investigating the effect of sTWEAK on osteoclastogenesis and furthermore the effect of sTWEAK on osteoblasts *in vitro*.

## Materials and methods

### Patients

This study was approved by the Human Ethics Committees of the University of Adelaide and The Repatriation General Hospital, and informed consent was obtained from all patients and healthy donors. RA patients fulfilled the 1987 revised criteria of the American College of Rheumatology (ACR) [19]. OA patients fulfilled the criteria by Altman and colleagues [20]. All patients with active RA had joint inflammation and patients with inactive RA were in remission after successful disease modifying anti-rheumatic drug (DMARD) treatment. Normal synovial tissues were obtained as previously described [21]. Synovial tissue was obtained from 39 patients (10 active RA, 9 inactive RA, 10 OA patients and 10 normal subjects) at the time of knee arthroscopy or total knee replacement surgery (OA patients) at the Rheumatology Unit, Repatriation General Hospital, South Australia. Characteristics of patients for IHC are summarised in Table 1.

**Table 1:** Demographic of patient for IHC TWEAK and Fn14 in synovial tissues

	Control	Active RA	Inactive RA	OA
**Gender (Female/Male)**	4/6	6/4	3/6	3/7
**Mean Age (years) (range)**	40.2 (25 to 58)	69.2 (31 to 86)	72.33 (60 to 79)	69.3 (55 to 77)
**Disease duration (months) (range)**	NA	3.1 (2 to 6)	21.77 (7 to 36)	NA
**Rheumatoid Factor**	NA	6/10	5/9	NA
**Erosions**	NA	2/10	2/9	NA
**C-Reactive protein median (mg/ml) (range)**	NA	67.5 (27.0 to 307.0)	6 (2.0 to 26.0)	NA
**Treatment**	NA	9 NSAIDs	5 im gold	6 NSAIDs
		1 prednisolone	2 methotrexate	3 NSAIDs
			1sulphasalazine	1 panadeine
			1 plaquenil	

NA, not available; NSAIDs: non steroidal anti-inflammatory drugs

Synovial fluid samples for ELISA were obtained from 17 active RA (5 female/12 male) and 16 OA (7 female/9 male) subjects. The mean age (± SEM) of the OA group was 66.40 ± 3.18 and of the active RA group was 63.19 ± 3.78.

### Immunohistochemical detection of TWEAK

TWEAK was detected using the previously described IHC method [22,23]. Briefly, tissue sections (5 μm) were deparaffinised, pre-treated with proteinase-K to unmask the antigen and treated with sodium azide in PBS (0.1% w/v) and H_2_O_2 _(0.3% v/v) to inhibit endogenous peroxidase. Sections were then incubated overnight with mouse anti-human TWEAK monoclonal antibody (MAb), P2D10 (15 μg/ml) [6]. Secondary antibody (HRP-conjugated goat anti-mouse IgG, DAKO, Botany, NSW, Australia) was then added, followed by HRP-conjugated swine anti-goat IgG (Biosource, Camarillo, CA, USA). The colour reaction was developed using AEC (3,9 aminoethylcarbazole from Sigma, St. Louis, MO, USA). Counterstaining was performed using Harris haematoxylin and lithium carbonate. An isotype-matched, non-binding control antibody (1D4.5, isotype IgG2a) at an identical IgG concentration or omission of the primary antibody, was used as a negative control. Tonsil tissues obtained at surgery were used as controls for lymphocytic staining.

### Immunohistochemical detection of Fn14

Fn14 was detected using previously published methods [24]. Briefly, sections were deparaffinised and pre-treated with 10 mM sodium citrate buffer (pH 6.0) at 95°C for 20 minutes. Endogenous peroxidase was then blocked using 0.3% v/v H_2_O_2 _in methanol solution. Blocking serum was applied, according to the manufacturer's instructions (Vectastain Universal Elite ABC kit, Vector Laboratories, Burlingame, CA, USA). Sections were then incubated with anti-human Fn14 MAb (ITEM1; Biolegend, San Diego, CA, USA) at 20 μg/ml overnight. Appropriate biotinylated secondary antibody was then added in the presence of 10% normal horse serum. Sections were then treated with avidin-biotin complex reagent before being stained with 3,3' Diaminobenzidine (DAB) (Vector Labs). Counter-staining and isotype-matched negative controls were performed as above for TWEAK staining.

### Dual immunohistochemistry

Double staining was performed to identify specific cell types expressing TWEAK using a previously published method [23]. Anti-TWEAK antibody was combined with MAbs for human cell surface markers: CD68 (macrophage; clone KP-1, Dako), CD22 (B lymphocyte; MAB1968, R&D Systems, Minneapolis, MN, USA), Tryptase G3 (mast cell; Cell Marque, Rocklin, CA, USA) and CD38 (plasma cells, BD Biosciences, Franklin Lakes, NJ, USA). After the single immunoperoxidase staining described above, the colour reaction was developed using an alkaline phosphatase reaction. Normal donkey serum (20% in PBS) was used to block non-specific binding, followed by incubation with cell surface marker antibodies overnight. Sections were incubated with AP-conjugated donkey anti-mouse IgG (Jackson Immuno Research, West Grove, PA, USA) as secondary antibody followed by incubation with mouse APAAP IgG (Dako, Botany, NSW, Australia). Colour was developed using a Fast Blue substrate. Using this method, immunoperoxidase stained cells were red, immuno-alkaline phosphatase stained cells were blue, while cells with co-expression were purple.

### Quantification of immunohistochemical staining

A semi-quantitative (SQA) scoring method was used to assess single immunohistochemical labelling, using a validated scoring system [22]. SQAs were analysed statistically by SPSS 11.5 software (SPSS Inc. Chicago, IL, USA) using non-parametric analysis. A *P-*value of less than 0.05 was considered to be significant.

### Detection of TWEAK mRNA in CD22^+ ^B Cells

Human peripheral blood mononuclear cells (PBMC) were prepared from normal buffy coat blood packs (Australian Red Cross Society, Adelaide, SA) on Ficoll gradients, as previously described [25]. CD22-expressing cells were isolated by fluorescence activated cell sorting (FACS) after staining with a mouse anti-human CD22 monoclonal antibody directly conjugated to phycoerythrin (anti-human CD22RPE, Clone 4KB128, Dako, Golstrup, Denmark), by a method essentially described previously [25]. CD22^+ ^or CD22^- ^cells were pelleted by centrifugation and resuspended in Trizol reagent. Total RNA and complementary DNA (cDNA) were prepared, and real-time reverse transcription polymerase chain reaction (RT-PCR) performed for the expression of TWEAK mRNA, as previously described [16].

### Detection of sTWEAK in synovial fluids

sTWEAK levels were measured following the manufacturer's instructions using a TWEAK instant ELISA kit (Bender MedSystem, Burlingame, CA, USA). Briefly, samples were added in duplicate to a 96-microwell plate, which had been pre-coated with antibody, capture antibody and secondary antibody and colour was developed using tetramethylbenzidine (TMB) substrate. The absorbance was read at 450 nm using Multiskan Ascent plate reader (Thermo Labsystems, Helsinki, Finland). A standard curve was generated from the control sera provided with the kit. The difference between the mean levels of sTWEAK in the two groups was analysed using an independent sample t test. A *P-*value of less than 0.05 was considered to be significant.

### Osteoclastogenesis assays in human PBMC and RAW 264.7 cells

Human CD14^+ ^PBMC were isolated by FACS, as described previously in detail [25]. The isolated cells were plated at 2 × 10^5 ^cells/well into a 96-microwell plate for tartrate resistant acid phosphatase (TRAP) staining or on whale dentine slices for resorption assays. Cells were cultured in αMEM medium containing 10% foetal calf serum (FCS) and 10 nM dexamethasone (Fauldings, Adelaide, SA, Australia) and rhM-CSF (25 ng/ml; Millipore, Temecula, CA, USA). Cultures received additional rhTWEAK and/or rhRANKL as indicated and were fed every three days. TRAP activity was assessed at 9 days and resorption was assessed by scanning electron microscopy (SEM) after 14 days, as described previously [25].

Murine RAW 264.7 cells were plated at 1 × 10^5 ^cells/well into 96-well plates, in the presence or absence of recombinant human (rh) Receptor Activator of Nuclear factor Kappa-B Ligand (RANKL) (100 ng/ml) (Millipore) and/or rhTWEAK (10 to 800 ng/ml). Cells were cultured for up to seven days in α-MEM medium containing 10% FCS and 10 nM 1α,25-dihydroxyvitamin D_3 _(1,25 D) [26] then stained for TRAP using a commercial kit (Sigma) at Day 7.

### Osteoblast assays

Human primary osteoblasts were cultured from cancellous bone samples obtained from patients undergoing total hip replacement surgery, as described previously [16]. Cells (10^5^/well) were seeded into wells of a six-well plate and cultured overnight in αMEM medium containing 10% v/v FCS. Cells were then cultured untreated or treated with recombinant human TWEAK (50 ng/ml) for three days [16]. Cells were enzymically removed and stained for STRO-1 [27] as previously described [28], and RANKL (MAB6261, R&D Systems) using an anti-mouse IgM-PE and anti-mouse IgG-FITC conjugate, respectively. Isotype-matched negative control antibodies 1A6.12 (IgM) and 1B5 (IgG_1_) were used to determine the level of background fluorescence for each fluorochrome and the compensation settings. Stained cells were analysed on a FACStar^PLUS ^flow cytometer (Becton Dickinson, Sunnyvale, CA, USA). The percentages of cells positive for either or both STRO-1 and RANKL were calculated.

## Results

### Immunohistochemistry

**Figure 1:**
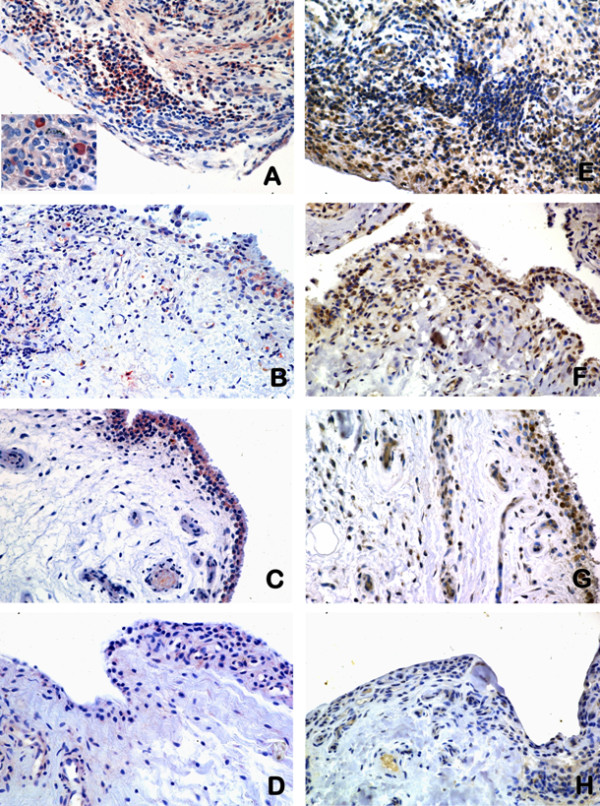
**Immuhistochemical staining of TWEAK and Fn14 in various synovia**. Expression of TWEAK (indicated by red, left) and Fn14 (indicated by brown, right) in active RA (**A, E**), inactive RA (**B, F**), OA (**C, G**) and normal synovial tissue (**D, H**). Insert in panel A is TWEAK expression by cells with resembling plasma cell-like morphology. All sections were counterstained with haematoxylin. Images were obtained using a 20× objective.

**Figure 2:**
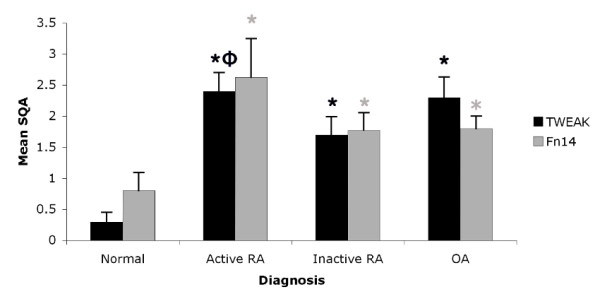
**SQA analysis of TWEAK and Fn14**. TWEAK and Fn14 expression in various synovial tissues as expressed by mean SQA ± SEM. Significant differences (*P *< 0.05) are indicated by * compared to normal and Φ compared to inactive RA synovial tissue.

Immunohistochemical staining demonstrated that TWEAK was expressed at significantly higher levels in synovial tissue from active RA, inactive RA and OA patients (Figure 1A-C, respectively) compared to normal controls (Figure 1D). The majority of cells expressing cytoplasmic TWEAK had either a macrophage-like morphology and were scattered in the synovial lining or appeared to be lymphocytes in the sub-lining region of active RA specimens. Significantly higher levels of TWEAK staining were observed in active RA compared to inactive RA synovial tissue (*P *< 0.05, Figures 1A, B and 2). In OA tissues (Figure 1C), TWEAK expression was confined mainly to the lining region with much weaker staining intensity compared to active RA synovial tissue. In addition, TWEAK was weakly expressed by cells lining the blood vessels, particularly in the synovial tissue from normal controls (Figure 1D).

Fn14 was highly expressed in synovia from active RA patients (Figure 1E) compared to other groups (Figure 1F-H). There was a statistically significant difference between Fn14 expression in normal tissue compared to diseased groups (Figure 2), but not between active RA and inactive RA groups. There was a strong correlation between the relative expression of TWEAK and Fn14 in synovial tissues (Kendall tau_ b test, *r *= 0.443, *P *= 0.001).

### Cell types expressing TWEAK and Fn14

**Figure 3:**
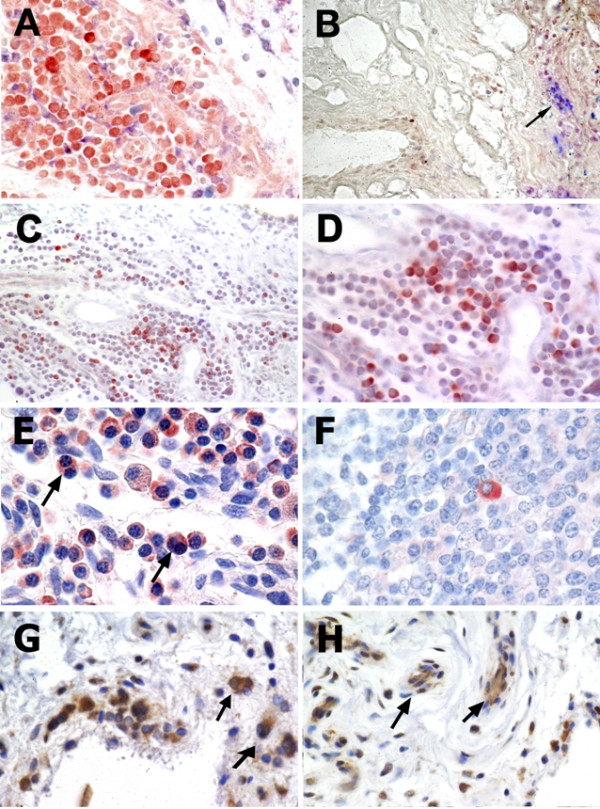
**Dual immunostaining for TWEAK and cell lineage markers and cells expressing Fn14**. Dual immunostaining for TWEAK (red) and CD68 (blue) in inflamed synovial tissue from a patient with active RA (**A**). Dual immunostaining for TWEAK (red) with CD38 (blue) with co-expression of TWEAK and CD38 (purple) indicated by arrow (**B**). **C**) and **D**) Dual immunostaining for TWEAK (red) with CD22 (blue). **E**) TWEAK expression (red) in multinucleated cells (indicated by arrows), and **F**) by plasma cells in tonsil tissue. Expression of Fn14 (brown) in multinucleated cells (**G**), and blood vessels of the synovial tissue (**H**), indicated by arrows. Sections shown in E, F, G, and H were counterstained with haematoxylin. Images shown in B and C were obtained with obj ×10; image shown in A obtained with obj ×20, D, F, G, H with obj ×40 and E with obj ×60.

In the active RA tissues a subset of the CD68-positive cells (macrophages) weakly expressed TWEAK (Figure 3A). In addition there is a subset of TWEAK positive cells which are positive for the plasma cell marker, CD38 (Figure 3B). However, the majority of cells strongly expressing TWEAK were positive for the B lymphocyte marker, CD22 (Figure 3C, D). TWEAK was not expressed by cells staining positive for the mast cell marker, Tryptase G, in any of the patient tissues (data not shown). On close inspection, some of the cells expressing TWEAK contained multiple nuclei (arrowed in Figure 3E). In the control tonsil tissue TWEAK was expressed by only a few B lymphocytes (Figure 3F). Due to the staining conditions required for the Fn14 MAb, dual labelling studies with this antibody were not possible. The majority of cells expressing Fn14 were similar in morphology to those expressing TWEAK (monocytes or lymphocytes). Close examination of the cells staining for Fn14 showed that some of these cells were also multinucleated cells (Figure 3G). Cells lining the small blood vessels also expressed Fn14. This was seen to some extent in all the tissues but was particularly prominent in the active RA tissue (Figure 3H).

**Figure 4:**
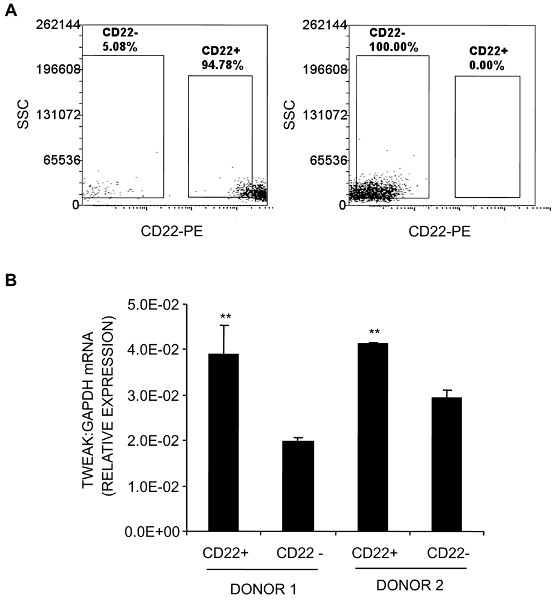
**TWEAK expression by PBMC**. PBMC from two healthy volunteers were sorted by FACS based on their expression of CD22, yielding CD22^+ ^and CD22^- ^populations of greater than 94% purity based on post-sort analysis (**A**). Isolated cells were then analysed for TWEAK mRNA expression relative to that of GAPDH, by real-time RT-PCR (**B**). Data shown are means of triplicate reactions ± SD. Differences in relative expression of TWEAK mRNA between CD22^+ ^and CD22^- ^populations were tested by Student's *t*-test (**P < 0.001).

Given that the clone used to detect TWEAK, P2D10, is function neutralising [6], it is unlikely that TWEAK-positivity was due to Fn14-positive cells binding soluble TWEAK. However, to confirm that B cells are a potential source of TWEAK, CD22-expressing or non-expressing cells were isolated by FACS and real-time RT-PCR performed for TWEAK mRNA expression. As shown in Figure 4, CD22^+ ^PBMC from two healthy volunteers expressed abundant TWEAK mRNA, relative to that of the housekeeping gene, GAPDH, and did so to a greater extent than the CD22^- ^fraction, consisting largely of T-cells and monocytes.

### TWEAK in synovial fluids from RA and OA

**Figure 5:**
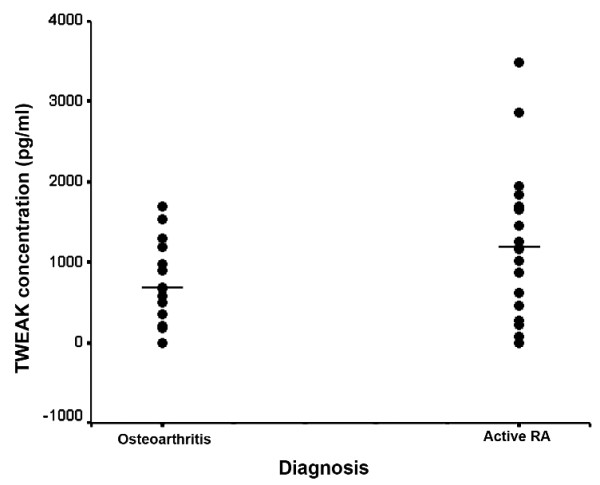
**Levels of soluble TWEAK in synovial fluids from patients with RA and OA**. Scatter plot of sTWEAK levels in the synovial fluids obtained from active RA (*n *= 17) and OA (*n *= 16). Horizontal lines reflect mean values of sTWEAK level in each group.

While not statistically significant, there was a trend (*P *= 0.079) for higher measurable levels of TWEAK protein in active RA synovial fluid (1,226 ± 235 pg/ml) than in the OA samples (713 ± 134 pg/ml) (Figure 5).

### Effect of soluble TWEAK on osteoclastogenesis *in vitro*

**Figure 6:**
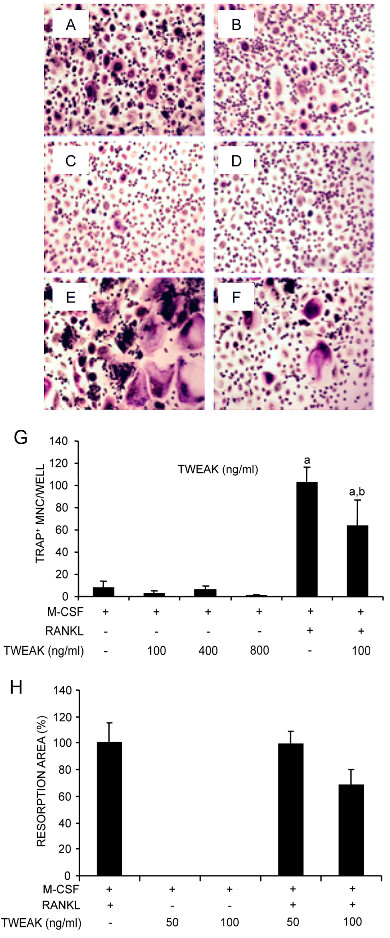
**Effect of soluble TWEAK on osteoclast formation and function**. Human unfractionated PBMC were cultured for nine days in medium containing rhM-CSF only (25 ng/ml) (**A**), M-CSF and rhTWEAK at 100 ng/ml (**B**), 400 ng/ml (**C**) or 800 ng/ml (**D**), M-CSF and rhRANKL (50 ng/ml) (**E**), or all of M-CSF (25 ng/ml), RANKL (50 ng/ml) and TWEAK (100 ng/ml) (**F**). Cultures were then fixed and stained for TRAP. (**G**) Multinucleated cells (MNC), containing >3 nuclei, positive for TRAP, were counted from quadruplicate wells. Data are expressed as means ± standard deviation. Significant differences were determined by one way analysis of variance (ANOVA) with Tukey post-hoc test: ^a ^indicates difference to M-CSF only control (*P *< 0.001) and ^b ^denotes difference to M-CSF+RANKL treatment (*P *< 0.05). (H) PBMC were seeded onto dentine slices in the presence of rhM-CSF (25 ng/ml) with the addition of rhRANKL (50 ng/ml) and/or rhTWEAK as indicated. Resorption was assessed after 14 days of culture by SEM and is expressed as the mean ± SD resorption expressed as a percentage of that measured for the RANKL/M-CSF control. Data shown are pooled from two independent experiments with resorption assessed for four dentine slices/treatment/donor. No significant differences were observed between RANKL treatments.

Recombinant human (rh) TWEAK at concentrations as high as 800 ng/ml in the presence of M-CSF did not stimulate osteoclast differentiation from unfractionated PBMC (Figure 6) or from fractionated CD14^+ ^human PBMC (not shown). Furthermore, RANKL/M-CSF osteoclastogenesis was inhibited rather than stimulated by rhTWEAK (Figure 6) as assessed by both TRAP staining and resorption pit formation on dentine slices (Figure 6). Identical results were obtained when murine RAW 264.7 cells were used as osteoclast precursors (data not shown).

### Effects of soluble TWEAK on cell surface RANKL expression

**Figure 7:**
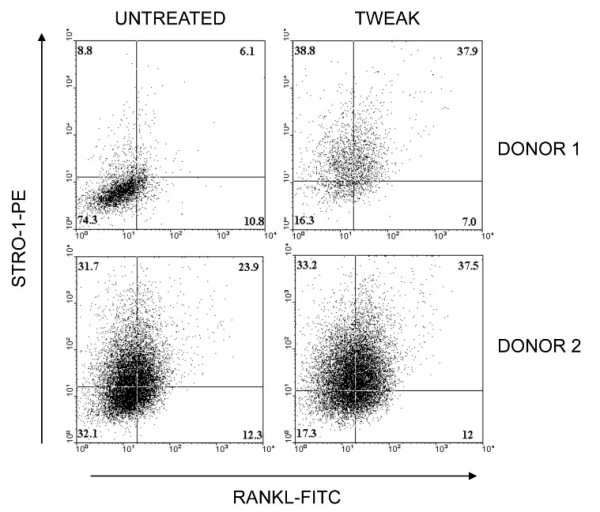
**Effect of soluble TWEAK on cell surface RANKL expression by STRO-1 osteoblast subpopulation**. Human primary osteoblasts were cultured untreated or treated with rhTWEAK (50 ng/ml) for three days, harvested by collagenase/dispase digestion and stained for STRO-1 and RANKL expression. Stained cells were analysed by FACS. The percentage of cells present in each of the four sub-populations is indicated. Data are presented for two independent donors' cells.

Treatment of human primary osteoblasts with rhTWEAK for three days resulted in the increased cell surface expression of RANKL. Furthermore, RANKL expression was induced on a population of osteoblasts expressing the immature osteoblast marker, STRO-1 (Figure 7). Together, these findings are consistent with the induction by TWEAK of a pro-osteoclastogenic osteoblastic phenotype.

## Discussion

The findings of this study are consistent with the growing evidence that TWEAK is a mediator of the joint destruction both in animal models of RA [10,11] and in human RA [12]. Recently, a number of cell types involved in the pathogenesis of RA have been reported to express TWEAK and its receptor, Fn14. While synovial fibroblasts from human patients with active RA have been reported to express TWEAK and Fn14 [14] another report demonstrated TWEAK expression by an unidentified CD45-positive haemopoietic cell population and Fn14 expression by both CD45-positive and -negative cells [12]. In contrast, van Kuijk and co-workers [14] reported TWEAK/Fn14 expression by macrophages but not by lymphocytes. The current study demonstrates that several cell types in RA synovial tissue express TWEAK, including CD68-positive macrophages, confirming the results of van Kuijk and co-workers [14]. Interestingly and in contrast to van Kuijk and co-workers, we observed that a sub-population of CD22-positive B lymphocytes and CD-38 positive plasma cells found in lymphocyte aggregates in the sub-lining synovial tissues expressed TWEAK. Our findings are consistent with those of Kraan and coworkers who found that the numbers of CD38^+ ^plasma cells and CD22^+ ^B cells in RA were the best discriminating markers when comparing RA to non-RA inflammatory synovial samples [22].

In the current study, TWEAK expression by plasma cells is in agreement with our recent finding that demonstrated the expression of TWEAK in bone marrow plasma cells in patients with multiple myeloma [18]. In that study, serum levels of TWEAK correlated with the serum bone resorption marker, β-crosslaps, and levels of the osteoclast recruitment chemokine, CXCL12, strongly implying a pathological role for plasma cell-derived TWEAK in bone erosion associated with multiple myeloma [18]. In the current study, we found that the TWEAK-positive B-Lymphocytes in the germinal centres of normal tonsil sections were rare, consistent with the infrequency of CD22^+ ^B cells at this site [29]. While the CD22^+^TWEAK^+ ^expressing B cells may be a specific population associated with the chronic inflammation seen in RA, CD22-positive B lymphocytes isolated from human normal peripheral blood also expressed abundant levels of TWEAK mRNA, suggesting that B-lineage cells are a source of TWEAK. Based on our findings in multiple myeloma [18] these cells may possibly also be associated with the bone erosion characteristic of RA. Interestingly, an immunotherapeutic approach that targets CD22, epratuzumab, has proved efficacious in the treatment of non-Hodgkin lymphoma, and in the autoimmune diseases, systemic lupus erythematosus and primary Sjögren's syndrome [30]. Our findings suggest that CD22 and CD38 are also potential therapeutic targets in RA.

We were unable to verify the cell types expressing Fn14 using dual label IHC for technical reasons; however, many of these cells also had the morphology of lymphocytes and monocytes and importantly our observation indicates that Fn14 was strongly expressed by multinucleated cells within the synovial tissue. We have previously demonstrated that these multinucleated cells were preosteoclasts [31]. It is well known that the active rheumatic joint contains an influx of osteoclast precursors [31]. This is also consistent with a recent study by our group demonstrating the expression of TWEAK and Fn14 by multinucleated cells in gingival tissues from chronic periodontitis patients [32]. This would suggest that TWEAK-Fn14 signalling may stimulate osteoclastogenesis and hence could contribute to the bone loss associated with chronic inflammatory diseases [10,11].

The observation that Fn14 is expressed by cells associated with blood vessels is consistent with the report that TWEAK regulates human endothelial cell proliferation, migration and survival *in vitro *[33,34]. Both TWEAK and Fn14 expression was associated with synovial tissue blood vessels suggesting that TWEAK has an autocrine effect on the angiogenesis seen in RA synovial tissue. Furthermore, this is consistent with the perivascular expression of TWEAK and Fn14 expression in RA and psoriatic synovial tissue [14].

A significant decrease in serum TWEAK has been reported in RA patients responding to treatment with etanercept [13,14] while persistent TWEAK/Fn14 expression was seen in a RA patient cohort receiving infliximab treatment [14] suggesting a role for TWEAK in modulation of disease activity. Our data demonstrated significantly lower expression of TWEAK in the inactive RA synovial tissues. In the majority of patients this followed successful non-TNF targeted DMARD treatment, suggesting that DMARDs may reduce TWEAK expression as seen in the etanercept-treated patients. Higher TWEAK expression in active RA was also reflected in the increased synovial fluid levels of sTWEAK compared with those in OA in our study. Based on this, we suggest that monitoring the sTWEAK levels in synovial fluids could possibly be another marker of disease progression.

It was previously reported that TWEAK stimulates the differentiation of RAW 264.7 monocyte/macrophage cells into functional osteoclasts, and did so in the absence of Fn14 expression [17]. However, we could not reproduce these findings *in vitro *using recombinant TWEAK to directly stimulate osteoclast formation from RAW 264.7 cells, or from human PBMC, either unfractionated or sorted on the basis of CD14 expression, a fraction shown previously to contain the osteoclast precursor [25,35]. Although the present study demonstrated that multinucleated cells expressed Fn14, our data indicate that the stimulation of osteoclast formation by TWEAK may not be due to a direct interaction with osteoclast precursors. However, we have demonstrated that stromal osteoblasts express Fn14 and are sensitive to the effects of TWEAK [10,16], including the induced expression of RANKL and CXCL12 mRNAs [18]. This supports an indirect role for TWEAK in recruiting and generating osteoclasts, via the osteoblast. In the current study we confirmed that TWEAK induced the expression of cell surface RANKL protein and did so in a population of human osteoblasts expressing the immature osteoblast cell surface marker, STRO-1, cells we have previously demonstrated are capable of supporting osteoclastogenesis [36]. This is reminiscent of our earlier study, in which RANKL mRNA expression was preferentially induced in STRO-1-positive osteoblasts in response to 1α,25-dihydroxyvitaminD_3 _[37]. In addition, we recently reported a novel role for TWEAK in the regulation of osteoblast differentiation [16] and showed that TWEAK, and the combination of TWEAK and TNFα, induced the expression of sclerostin, a negative regulator of bone formation [38], and as we recently demonstrated [39], human osteoblast differentiation and matrix mineralization. Thus, the elevated TWEAK expression we see in RA may not only stimulate bone resorption by osteoclasts by induction of RANKL on osteoblasts but also inhibit new bone formation, contributing to the reduction in bone mass observed in inflammatory bone disease [40].

## Conclusions

The current study found high levels of TWEAK expression in synovial tissues and synovial fluids from patients with active RA. Together with its ability to induce cell surface expression of RANKL by osteoblasts and possibly induce activation of B cells into plasma cells we postulate that TWEAK in this context would be pro-osteoclastogenic and would thus contribute to the bone loss associated with this chronic inflammatory disease. These findings support the concept that TWEAK should be considered as a therapeutic target in RA.

## Abbreviations

ACR: American College of Rheumatology; AEC: 3,9 aminoethylcarbazole; CIA: collagen induced arthritis; DAB: diaminobenzidine; DAS28: disease activity score; DMARD: disease modifying anti-rheumatic drug; FACS: fluorescence activated cell sorting; FCS: foetal calf serum; HRP: horse raddish peroxidase; HUVEC: human umbilical vein endothelial cells; IHC: immunohistochemistry; MAb: monoclonal antibody; MAPK: mitogen-activated protein kinases; MMP: matrix metalloproteinase; OA: osteoarthritis; PBMC: peripheral blood mononuclear cells; PBS: phosphate buffered saline; RA: rheumatoid arthritis; RANKL: Receptor activator of NF Kappa B Ligand; rh: recombinant human; SQA: semi-quantitative assessment; TMB: tetramethylbenzidine; TNF: Tumor necrosis factor; TRAP: tartrate resistant acid phosphatase; TWEAK: TNF-like weak inducer of apoptosis.

## Competing interests

The authors declare that they have no competing interests.

## Authors' contributions

AD played a major role in manuscript preparation, experimental work, statistical analysis and interpretation and MS directed patient recruitment and assessment and critical revision of the manuscript. TC carried out ELISA assays and analysis of ELISA data and wrote the manuscript. CAH and CV were substantially involved in *in vitro *assays. ACWZ was involved in experimental design and critical analysis of the manuscript. TSZ and DMF were involved in critical analysis of the manuscript. HMW. identified appropriate patient tissues available for the study. GJA initiated the project and was substantially involved in experimental design and critical analysis of the manuscript. DRH coordinated the project and was involved in critical revision of the manuscript.
